# Epstein-Barr Virus-Positive Lymphoma-Associated Hemophagocytic Syndrome: A Retrospective, Single-Center Study of 51 Patients

**DOI:** 10.3389/fimmu.2022.882589

**Published:** 2022-04-11

**Authors:** Ailin Zhao, Jinrong Yang, Meng Li, Linfeng Li, Xinai Gan, Jie Wang, He Li, Kai Shen, Yunfan Yang, Ting Niu

**Affiliations:** Department of Hematology, West China Hospital, Sichuan University, Chengdu, Sichuan, China

**Keywords:** epstein-barr virus, lymphoma, hemophagocytic syndrome, overall response rate, overall survival

## Abstract

**Purpose:**

To investigate clinical characteristics, management, and prognosis of Epstein-Barr virus (EBV)-positive lymphoma-associated hemophagocytic syndrome (LAHS) patients in real-world practice.

**Methods:**

This was a retrospective, single-center cohort study. EBV-positive LAHS patients diagnosed from January 2010 to December 2021 in our center were enrolled. Clinical characteristics, treatment, overall response rate (ORR), and overall survival (OS) were investigated. Univariate and multivariate analysis of potential factors were conducted.

**Results:**

Of the 51 patients, 44 were T/NK cell lymphoma; five were B cell lymphoma; two were Hodgkin lymphoma. EBV-positive T/NK cell LAHS patients were significantly younger and showed lower fibrinogen levels and C-reactive protein levels than EBV-positive B cell LAHS patients (*P*=0.033, *P*=0.000, and *P*=0.004, respectively). Combined treatment of anti-hemophagocytic lymphohistiocytosis (HLH) and anti-lymphoma treatment was conducted in 24 patients; anti-HLH treatment was conducted in 18 patients; anti-lymphoma treatment was conducted in three patients; glucocorticoid treatment was conducted in one patient. ORR was 47.8%, and the median OS was 61 (95% confidence interval 47.9-74.1) days for overall patients. Patients who received anti-HLH treatment and turned to anti-lymphoma treatment early displayed higher ORR and OS than those of anti-HLH patients (*P*=0.103, and *P*=0.003, respectively). Elevated alanine aminotransferase level was the independent risk factor of EBV-positive LAHS prognosis.

**Conclusions:**

Prognosis of EBV-positive LAHS patients was poor. Anti-lymphoma treatment should be initiated as soon as HLH was rapidly controlled.

## Introduction

Epstein-Barr virus (EBV) is a double-stranded DNA herpesvirus, whose infection is prevalent in 95% of the world’s population (1). EBV mainly infects B lymphocytes due to interaction between viral glycoprotein gp350 and CD21 receptor, with memory B cells serving as EBV reservoir (2). Besides B cells, EBV can also infect T and NK cells. EBV-associated lymphoproliferative disorders (LPDs) include various diseases, ranging from reactive disorders to lymphoma. The majority of EBV-associated lymphoma displays type II EBV latency, which expresses five nuclear antigens (EBNA1, 3A–C and LP) and three membrane proteins (LMP1, LMP2A and LMP2B), except EBNA2, one of the dominant transforming proteins. A minority of EBV-associated lymphoma, for example, Burkitt lymphoma, shows type I EBV latency, which only expresses EBNA1, regulating episomal EBV genome replication (3). Immune escape and immunodeficiency are essential to EBV-associated lymphomagenesis, depending on different subtypes of lymphoma and immune status of patients. In EBV^+^ Hodgkin lymphoma (HL) and EBV^+^ nodal diffuse large B cell lymphoma (DLBCL) young patients, immune escape plays an important part in pathogenesis, as evidenced by recurrent alterations in *PD-L1* and *PD-L2* (4, 5). The extent of PD-L1 expression was significantly related to EBV positivity (6). However, in EBV^+^ peripheral T cell lymphoma (PTCL), immunodeficiency leads to gradual impairment of T-cell antigenic repertoire, and the chronic inflammatory environment brings free radicals triggering lymphomagenesis (7, 8).

Hemophagocytic syndrome, also named hemophagocytic lymphohistiocytosis (HLH), is a rare but lethal hyperinflammatory syndrome. Aberrant activation of lymphocytes, monocytes, and macrophages leads to substantial release of inflammatory cytokines. HLH typically manifests itself as fever, hepatosplenomegaly, and multiple organ dysfunction syndrome (MODS). HLH can be categorized into primary HLH and secondary HLH. Primary HLH is characterized by early onset and cytotoxicity-related gene mutations. Secondary HLH mainly attacks adult patients secondary to malignancy, infection, autoimmune disease, pregnancy, and other triggers, with the incidence of 0.125 per 100,000 per year (9). Lymphoma-associated hemophagocytic syndrome (LAHS) is the most common type of secondary HLH. An epidemiological investigation of all HLH cases in China was conducted in 2019. In the general Chinese population, the incidence of LAHS was 0.014/100,000 per year (10). HLH can occur not only upon lymphoma onset, but also during relapse or advanced stage of lymphoma. Clinical features of LAHS largely overlap with those of other types of HLH, sepsis, and MODS, which could impede in-time diagnosis and treatment. Prognosis of LAHS patients is poor, with a median overall survival (OS) of 28 to 36 days (11, 12). Though EBV-positive LAHS has been described in case reports (13–15), comprehensive investigation of clinical characteristics and outcome of the EBV-positive LAHS cohort is rare. Thus, this study is a single-center, retrospective, cohort study to explore clinical features and prognosis of EBV-positive LAHS patients.

## Materials and Methods

### Participants and Treatment

Patients consecutively diagnosed as EBV-positive LAHS from January, 2010 to December, 2021 at West China Hospital, Sichuan University were enrolled (16, 17). EBV positivity was assessed by EBER *in situ* hybridization. Besides antiviral treatment, induction treatment was divided into anti-HLH treatment, anti-lymphoma treatment, and the combination of both. Anti-HLH treatment consisted of HLH-1994 regimen, HLH-2004 regimen, and GED regimen (gemcitabine-etoposide-dexamethasone). Anti-lymphoma treatment was chemotherapy depending on different subtypes of lymphoma. Treatment response was evaluated according to the following conditions. Complete response (CR) required disappearance of all HLH-related symptoms, and HLH-related lab findings returning to normal range, including ferritin, soluble CD25 (sCD25), complete blood count, triglyceride, alanine aminotransferase (ALT), etc. Consciousness should recover to normal if central nervous system was involved. Partial response (PR) required normal body temperature and improvement by over 25% in at least two symptoms or lab findings, including sCD25 decrease by at least one third, ferritin and triglyceride decrease by at least 25%, ALT decrease by at least 50% if formerly over 400U/L, and neutrophil increase by 100% without transfusion. If neutrophil counts were formerly less than 0.5×10^9^/L, they should exceed 0.5×10^9^/L after treatment. If neutrophil counts were formerly over 0.5×10^9^/L but less than 2×10^9^/L, they should exceed 2×10^9^/L after treatment. No response (NR) was defined as being unable to reach CR or PR. Overall response rate (ORR) was calculated as (CR+PR)/total patients × 100%. OS was defined as the interval between HLH diagnosis and all-cause death. The patients were followed up until December, 2021. This study was conducted with the approval of the West China Hospital ethics committee, according to Declaration of Helsinki.

### Statistical Analysis

Statistical analysis was conducted with SPSS 23.0 and GraphPad Prism 7.0 software. Measurable data with normal distribution and skewed distribution were evaluated with *t* test and *U* test, respectively. Enumeration data were evaluated with *χ²* test. Survival analysis was assessed with Kaplan-Meier methods, and compared with Log rank test. Univariate and multivariate analysis of prognostic predictors was conducted with Cox proportional hazard model. It was regarded as statistically significant with *P*<0.05.

## Results

### Baseline Characteristics

Fifty-one EBV-positive LAHS patients were enrolled in this study, including 35 (68.6%) male patients and 16 (31.4%) female patients. Median age at HLH diagnosis was 37 (13-64) years. Three (5.9%) patients were newly diagnosed as lymphoma, while 48 (94.1%) patients were relapsed/refractory lymphoma cases. Clinical characteristics of these patients at baseline were listed in **Table 1**. The total cohort consisted of 44 (86.3%) T/NK cell lymphoma cases, five (9.8%) B cell lymphoma cases, and two (3.9%) HL cases. Regarding T/NK cell lymphoma, extranodal NK/T-cell lymphoma (ENKL), nasal type (22/51, 43.1%) and aggressive natural killer cell leukemia (ANKL) (13/51, 25.5%) were the dominant subtypes. Besides, PTCL, not otherwise specified (PTCL, NOS) (4/51, 7.8%), angioimmunoblastic T cell lymphoma (AITL) (3/51, 5.9%), anaplastic large cell lymphoma (ALCL) (1/51, 2.0%), and systemic EBV-positive T-cell lymphoma of childhood (1/51, 2.0%) were also discovered. B cell lymphoma group included four (7.8%) DLBCL cases and one (2.0%) lymphoplasmacytic lymphoma (LPL) case. The majority of (94.1%) EBV-positive LAHS patients were at Ann Arbor stage III-IV. In terms of clinical manifestations, fever was present in all patients. Splenomegaly, serous effusion, hepatomegaly, edema, and jaundice were seen in 42 (82.4%), 28 (54.9%), 22 (43.1%), 12 (23.5%), and 10 (19.6%) patients, respectively. Hemophagocytosis phenomena were discovered in 24 (47.1%) patients.

**Table 1:** Clinical characteristics of EBV-positive LAHS patients at baseline.

Characteristics	n (%)
Sex	
Male	35 (68.6)
Female	16 (31.4)
Age at HLH diagnosis, median (range)	37 (13-64)
Lymphoma subtype	
T/NK cell lymphoma	44 (86.3)
Extranodal NK/T-cell lymphoma, nasal type	22 (43.1)
Aggressive natural killer cell leukemia	13 (25.5)
Peripheral T cell lymphoma, not otherwise specified	4 (7.8)
Angioimmunoblastic T cell lymphoma	3 (5.9)
Anaplastic large cell lymphoma	1 (2.0)
Systemic EBV-positive T-cell lymphoma of childhood	1 (2.0)
B cell lymphoma	5 (9.8)
Diffuse large B cell lymphoma	4 (7.8)
Lymphoplasmacytic lymphoma	1(2.0)
Hodgkin lymphoma	2 (3.9)
Lymphoma stage	
I-II stage	3 (5.9)
III-IV stage	48 (94.1)
Clinical manifestations	
Fever	51 (100)
Splenomegaly	42 (82.4)
Serous effusion	28 (54.9)
Hepatomegaly	22 (43.1)
Edema	12 (23.5)
Jaundice	10 (19.6)
Lab test, median (range)	
Hemoglobin (g/L)	96 (44-132)
Neutrophil (×10^9^/L)	1.53 (0.03-12.58)
Platelet (×10^9^/L)	45 (7-264)
Alanine aminotransferase (U/L)	87 (10-597)
Aspartate aminotransferase (U/L)	108 (15-936)
Total bilirubin (μmol/L)	20.55 (5.20-178.90)
Albumin (g/L)	30.55 (19.0-55.0)
Lactate dehydrogenase (U/L)	758.5 (108-3346)
Triglyceride (mmol/L)	2.885 (0.90-7.66)
Fibrinogen (g/L)	1.60 (0.50-6.57)
Ferritin (ng/mL), n=47	2799 (314-161681)
sCD25 (pg/mL), n=38	8547.5 (1455-36685)
Hemophagocytosis phenomenon	24 (47.1)
Plasma EBV-DNA (copies/mL)	145000 (647-744000000)

LAHS, lymphoma-associated hemophagocytic syndrome.

Baseline characteristics of EBV-positive T/NK cell LAHS patients were compared to those of EBV-positive B cell LAHS patients. T/NK cell LAHS patients were significantly younger than B cell LAHS patients upon HLH diagnosis (34 v.s. 47 years, *P*=0.033). Fibrinogen level and C-reactive protein (CRP) level were significantly lower in T/NK cell LAHS patients than B cell LAHS patients (*P*=0.000 and *P*=0.004, respectively). Plasma EBV DNA level tended to be higher in B cell LAHS patients than in T/NK cell LAHS patients, but the difference was not significant. There was no significant difference in sex, IPI score, ECOG score, clinical manifestations, complete blood count, liver and kidney function, triglyceride, ferritin, sCD25, hemophagocytosis phenomenon, and the occurrence order of lymphoma and HLH between EBV-positive T/NK cell LAHS patients and EBV-positive B cell LAHS patients (**Table 2**).

**Table 2:** Comparison of baseline characteristics between EBV-positive T/NK cell LAHS patients and EBV-positive B cell LAHS patients.

Characteristics, n (%)	T/NK cell LAHS (n=44)	B cell LAHS (n=5)	*P*
Sex			1.000
Male	30 (68.2)	3 (60.0)	
Female	14 (31.8)	2 (40.0)	
Age at HLH diagnosis, y, median (range)	34 (13-64)	47 (44-63)	0.033
IPI score			0.117
0-1	0	0	
2-3	23 (52.3)	5 (100)	
4-5	21 (47.7)	0	
ECOG score			0.540
0-2	24 (54.5)	4 (80.0)	
3-5	20 (45.5)	1 (20.0)	
Manifestations			
Splenomegaly	36 (81.8)	4 (80.0)	1.000
Serous effusion	24 (54.5)	3 (60.0)	1.000
Hepatomegaly	19 (43.2)	2 (40.0)	1.000
Edema	12 (27.3)	0	0.427
Jaundice	10 (22.7)	0	0.542
Lab tests, median (range)			
Hemoglobin (g/L)	97.5 (44-132)	101 (74-117)	0.870
Neutrophil (×10^9^/L)	1.30 (0.03-12.58)	1.89 (0.83-4.72)	0.714
Platelet (×10^9^/L)	45 (7-264)	44 (35-128)	0.682
Alanine aminotransferase (U/L)	93 (13-597)	81 (10-146)	0.428
Aspartate aminotransferase (U/L)	111 (17-936)	97 (15-297)	0.561
Total bilirubin (μmol/L)	22.5 (5.2-178.9)	19.8 (16.6-67)	0.714
Albumin (g/L)	30.55 (19.0-55.0)	31.3 (20.6-35.4)	0.665
Lactate dehydrogenase (U/L)	771 (108-3346)	577 (399-1167)	0.409
Triglyceride (mmol/L)	3.37 (0.93-7.66)	2.08 (0.90-3.87)	0.131
Fibrinogen (g/L)	1.32 (0.5-6.2)	4.15 (2.36-6.57)	0.000
Ferritin (ng/mL), n=47	2861.5 (314-161681)	2431.8 (863.7-20063)	0.578
sCD25 (pg/mL), n=38	7699 (1455-36685)	8714 (5396-21578)	0.758
C-reactive protein (mg/L)	35.05 (2.32-167)	122 (86.2-228)	0.004
Hemophagocytosis phenomenon	20 (45.5)	2 (40.0)	1.000
Plasma EBV-DNA (copies/mL)	142000(654-3200000)	278000(7610-744000000)	0.375
Occurrence order of lymphoma and HLH			0.632
HLH prior to lymphoma	4 (9.1)	0	
lymphoma prior to HLH	7 (15.9)	1 (20.0)	
Simultaneous occurrence	33 (75.0)	4 (80.0)	

HLH, hemophagocytic lymphohistiocytosis; LAHS, lymphoma-associated hemophagocytic syndrome.

We also compared EBV-positive LAHS patients and EBV-negative LAHS patients who were consecutively diagnosed in our department during the same period of time. EBV-positive LAHS patients (median 37 years, range 13-64 years) were significantly younger at HLH diagnosis than EBV-negative LAHS patients (median 42 years, range 13-90 years, *P*=0.049). EBV-positive LAHS patients displayed significantly higher hemoglobin and triglyceride levels at baseline than EBV-negative LAHS patients (*P*=0.008, and *P*=0.007, respectively). T/NK-cell lymphoma tended to account for a higher proportion of EBV-positive LAHS patients (86.3%) than EBV-negative LAHS patients (69.8%), but the difference was insignificant (**Table S1**).

### Treatment and Response

**Figure 1:**
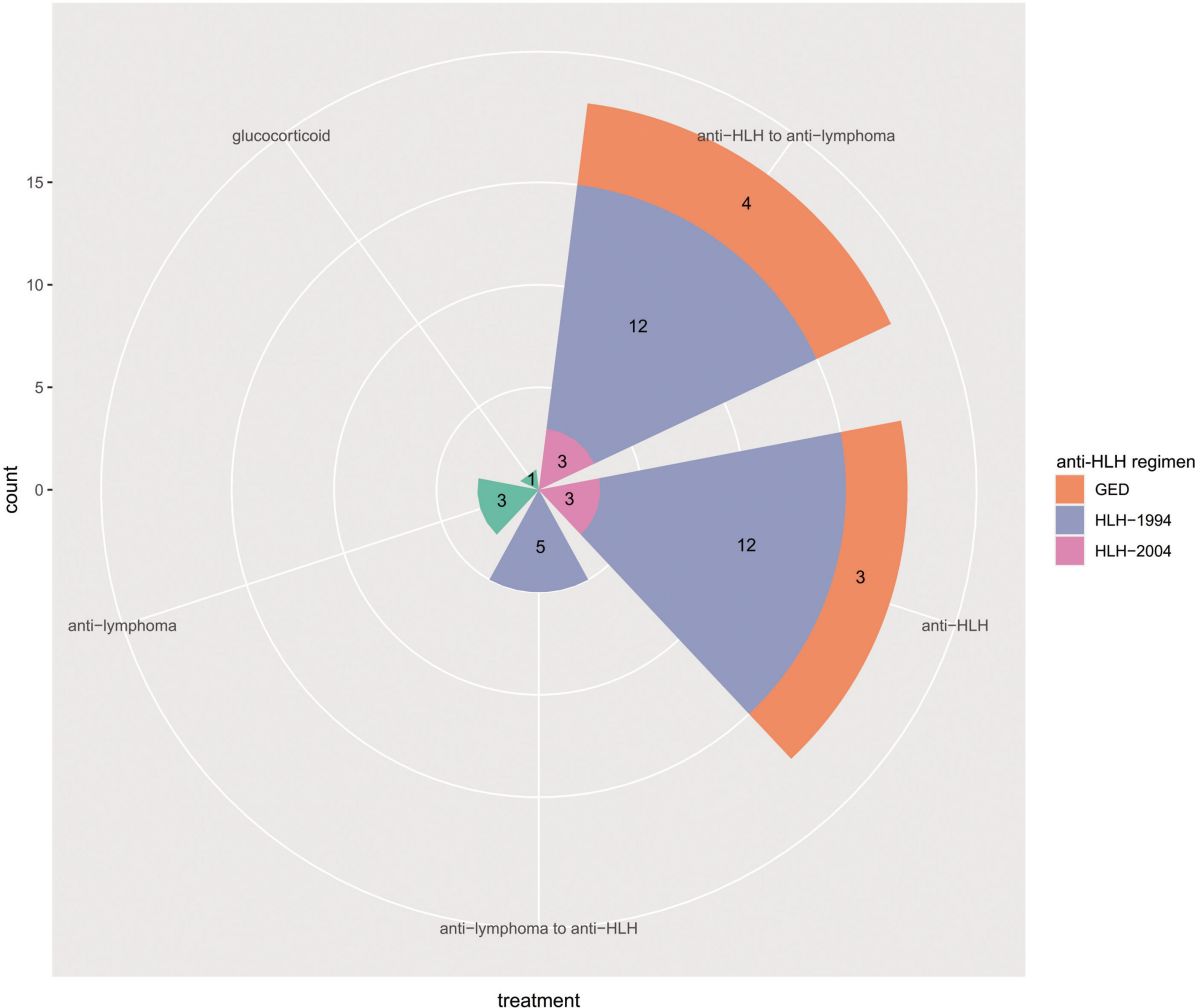
Treatment of EBV-positive lymphoma-associated hemophagocytic syndrome patients.

Of the 51 patients, three patients only received antiviral treatment in our department, and two patients initiated HLH-1994 regimen but died in three days. After excluding these five patients, the remaining 46 patients were evaluable for treatment response. Treatment was divided into combined treatment group, anti-HLH treatment group, anti-lymphoma treatment group, and glucocorticoid treatment group (**Figure 1**). Combined treatment of anti-HLH regimen and anti-lymphoma chemotherapy was conducted in 24 (52.2%) patients. Nineteen (41.3%) patients first underwent anti-HLH treatment of 3 (1-9) weeks, and then turned to anti-lymphoma chemotherapy. HLH-1994 regimen was conducted in 12 (26.1) patients. GED regimen was given to four (8.7%) patients, and HLH-2004 regimen was performed in three (6.5%) patients. Median number of chemotherapy cycles was 2 (1-8). The detailed anti-lymphoma chemotherapy was listed in **Table 3**. Another five (10.9%) patients first received anti-lymphoma chemotherapy and turned to HLH-1994 regimen subsequently. These patients were all ENKL patients, and the detailed chemotherapy was also listed in **Table 3**. The median number of chemotherapy cycles was 1 (1-3). The median weeks of HLH-1994 treatment was 4 (2-4).

**Table 3:** Combined treatment of anti-HLH and anti-lymphoma regimen in EBV-positive LAHS patients.

Anti-HLH regimen prior to anti-lymphoma chemotherapy	n (%)
ENKL, n=8	
GLIDE	6 (75%)
SMILE	1 (12.5%)
ECHOP	1 (12.5%)
ANKL, n=5	
GLIDE	4 (80%)
L-GIFoX	1 (20%)
PTCL, NOS, n=2	
CHOP	2 (100%)
AITL, n=1	
Chi-CHOP	1 (100%)
DLBCL, n=1	
RCHOP	1 (100%)
HL, n=2	
ABVD	2 (100%)
**Anti-lymphoma chemotherapy prior to anti-HLH regimen**	
ENKL, n=5	
GLIDE	2 (40%)
VDLP	2 (40%)
VLP	1 (20%)

AITL, angioimmunoblastic T cell lymphoma; ANKL, aggressive natural killer cell leukemia; DLBCL, diffuse large B cell lymphoma; ENKL, extranodal NK/T-cell lymphoma, nasal type; HL, Hodgkin lymphoma; HLH, hemophagocytic lymphohistiocytosis; LAHS, lymphoma-associated hemophagocytic syndrome; PTCL, Peripheral T cell lymphoma.

In the 18 (39.1%) patients who only received anti-HLH treatment, the median weeks of induction treatment was 4 (1-8). The majority of (12/18, 66.7%) patients received HLH-1994 regimen. HLH-2004 regimen (3/18, 16.7%) and GED regimen (3/18, 16.7%) were conducted in three patients each. Three (6.5%) patients only received anti-lymphoma chemotherapy. One ENKL patient and one ANKL patient received GLIDE regimen, and one DLBCL patient received chidamide-RCHOP regimen. Median number of chemotherapy cycles was 3 (2-8). One (2.2%) patient only received glucocorticoid treatment.

For patients who were at higher risk or failed to achieve CR with a poor control of EBV infection, consolidation treatment was conducted, which included PD-1 monoclonal antibody treatment, chidamide treatment, and autologous or allogenic stem cell transplantation. For patients whose biopsy revealed positive PD-L1 staining of tumor cells, especially HL patients and DLBCL patients, PD-1 monoclonal antibody treatment was conducted. For patients who displayed aberrant epigenetic alterations, especially PTCL patients and double expression DLBCL patients, chidamide treatment was conducted. For young, fit, and high-risk patients with sufficient stem cells collected who were willing to receive transplantation, autologous or allogenic stem cell transplantation was conducted. Overall, seven (15.2%) patients underwent consolidation therapy. Three (6.5%) patients (one HL case, one DLBCL case, and one ENKL case) received PD-1 monoclonal antibody treatment, of whom the DLBCL patient also received autologous stem cell transplantation after PD-1 monoclonal antibody treatment. Two (4.3%) patients (one ENKL case and the DLBCL patient who received PD-1 monoclonal antibody treatment) received autologous stem cell transplantation. One (2.2%) ANKL patient received allogenic stem cell transplantation. Two (4.3%) AITL patients received chidamide treatment.

**Figure 2:**
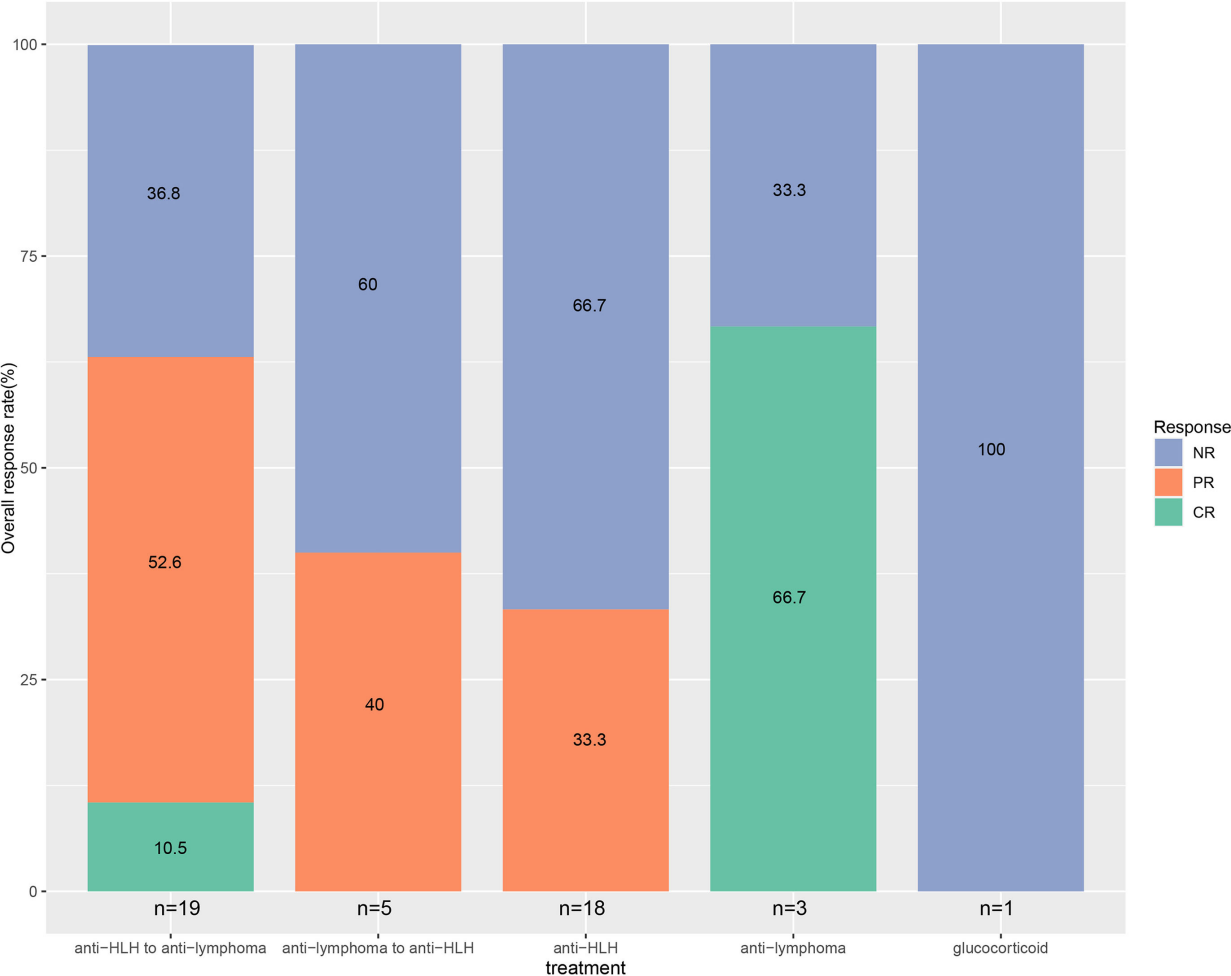
Response rate of EBV-positive lymphoma-associated hemophagocytic syndrome patients.

Of the 46 evaluable patients, CR was achieved in four (8.7%) patients, and PR was achieved in 18 (39.1%) patients. Twenty-four (52.2%) patients remained NR. ORR was 47.8%. In the combined treatment group, CR was reached in two (8.3%) patients, and PR was reached in 12 (50%) patients. NR was present in 10 (41.7%) patients. ORR was 58.3%. For patients who first received anti-HLH treatment and turned to anti-lymphoma chemotherapy subsequently, CR was achieved in two (10.5%) patients, and PR was achieved in 10 (52.6%) patients. Seven (36.8%) patients remained NR. ORR was 63.2%. For those who first received anti-lymphoma chemotherapy and turned to anti-HLH treatment subsequently, PR was achieved in two (40%) patients, and three (60%) patients remained NR. ORR was 40%. In anti-HLH treatment group, six (33.3%) patients achieved PR and twelve (66.7%) patients remained NR. ORR was 33.3% for the total anti-HLH treatment group, the HLH-1994 regimen subgroup, the HLH-2004 regimen subgroup, and the GED regimen subgroup. In anti-lymphoma treatment group, CR was achieved in two (66.7%) patients while one (33.3%) patient remained NR. ORR was 66.7%. One patient only received glucocorticoid treatment but remained NR. Patients who first received anti-HLH treatment and turned to anti-lymphoma treatment subsequently tended to show a higher ORR than those who received anti-HLH treatment only, but the difference was not significant (63.2% v.s. 33.3%, *P*=0.103). There was no significant difference in ORR among different treatment groups (**Figure 2**).

At baseline, plasma EBV-DNA of all patients was positive (median 145000 copies/mL, range 647-744000000 copies/mL). After induction treatment, plasma EBV-DNA decreased significantly in all patients but one, who was a ANKL patient only receiving anti-HLH treatment but remaining NR (median 643.5 copies/mL, range 0-88800 copies/mL). Plasma EBV-DNA of ten patients after treatment was negative. Another three patients had a plasma EBV-DNA level lower than 50 copies/mL after treatment. There was no significant difference in plasma EBV-DNA after treatment between T/NK-cell LAHS (median 623 copies/mL, range 0-88800 copies/mL) and B-cell LAHS patients (median 14835 copies/mL, range 0-32300 copies/mL, *P*=0.540). In terms of treatment group, median plasma EBV-DNA of patients after treatment in combined treatment group and anti-HLH group was 272.5 copies/mL (range 0-64800 copies/mL), and 3130 copies/mL (range 0-33800 copies/mL), respectively. For the three patients in anti-lymphoma group, median plasma EBV-DNA of patients after treatment was 0 copies/mL (range 0-50 copies/mL). For the only patient in glucocorticoid group, his plasma EBV-DNA level decreased from 158000 copies/mL to 88800 copies/mL after treatment. There was no significant difference in plasma EBV-DNA level after treatment among treatment groups.

Regarding EBV-positive T/NK cell LAHS patients, three patients only received antiviral treatment in our department, and one patient initiated HLH-1994 regimen but died in three days. After excluding these patients, 40 patients were evaluable for treatment response. Twenty-one (52.5%) patients received combined treatment. Sixteen (40%) patients first underwent anti-HLH treatment of 3.5 (1-9) weeks, and then turned to anti-lymphoma chemotherapy. Median number of chemotherapy cycles was 1 (1-8). Another five (12.5%) patients first received anti-lymphoma chemotherapy and turned to HLH-1994 regimen subsequently. The median number of chemotherapy cycles was 1 (1-3). The median weeks of HLH-1994 treatment was 4 (2-4). In the 16 (40%) patients who only received anti-HLH treatment, the median weeks of induction treatment was 4 (1-8). Two (5%) patients only received anti-lymphoma chemotherapy. Median number of chemotherapy cycles was 2.5 (2-3). One (2.5%) patient only received glucocorticoid treatment.

Of the 40 evaluable EBV-positive T/NK cell LAHS patients, CR was achieved in three (7.5%) patients, and PR was achieved in 14 (35%) patients. Twenty-three (57.5%) patients remained NR. ORR was 42.5%. In the combined treatment group, CR was reached in two (9.5%) patients, and PR was reached in nine (42.9%) patients. NR was present in 10 (47.6%) patients. ORR was 52.4%. For patients who first received anti-HLH treatment and turned to anti-lymphoma chemotherapy subsequently, CR was achieved in two (12.5%) patients, and PR was achieved in seven (43.75%) patients. Seven (43.75%) patients remained NR. ORR was 56.25%. For those who first received anti-lymphoma chemotherapy and turned to anti-HLH treatment subsequently, PR was achieved in two (40%) patients, and three (60%) patients remained NR. ORR was 40%. In anti-HLH treatment group, five (31.25%) patients achieved PR and 11 (68.75%) patients remained NR. ORR was 31.25%. In anti-lymphoma treatment group, CR was achieved in one (50%) patient while one (50%) patient remained NR. ORR was 50%. One patient only received glucocorticoid treatment but remained NR. There was no significant difference in ORR among different treatment groups.

We also compared treatment and response of EBV-positive LAHS patients and EBV-negative LAHS patients in our department. Treatment choice for these patients was significantly different, with a higher proportion of EBV-positive LAHS patients (52.2%) receiving combined treatment than EBV-negative LAHS patients (31.4%, *P*=0.033, **Table S2**). The ORR of EBV-positive LAHS patients (47.8%) was similar to that of EBV-negative LAHS patients (51.4%). The ORR of EBV-negative LAHS patients who received combined treatment (72.7%) tended to be higher than that of EBV-positive LAHS patients (58.3%), especially for those who first underwent anti-HLH treatment and turned to anti-lymphoma treatment subsequently (80% v.s. 63.2%), but the difference was insignificant (**Table S3**).

### Survival Outcome

**Figure 3:**
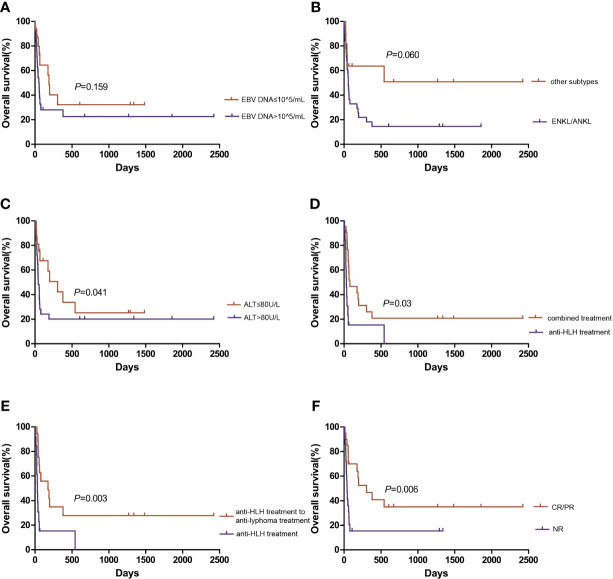
Overall survival of EBV-positive lymphoma-associated hemophagocytic syndrome patients. Comparison of overall survival based on **(A)** plasma EBV DNA level, **(B)** lymphoma subtype, and **(C)** alanine aminotransferase level. **(D)** Comparison of overall survival between combined treatment and anti-HLH treatment. **(E)** Comparison of overall survival between anti-HLH to anti-lymphoma treatment and anti-HLH treatment. **(F)** Comparison of overall survival based on treatment response. ALT, alanine aminotransferase; ANKL, aggressive natural killer cell leukemia; CR, complete response; ENKL, extranodal NK/T-cell lymphoma, nasal type; HLH, hemophagocytic lymphohistiocytosis; NR, no response; PR, partial response.

With a median follow-up of 79.5 (10-605) days, the median OS of the total cohort was 61 (95% CI 47.9-74.1) days. The median OS of EBV-positive NK/T cell LAHS patients was 65 (95% CI 52.7-77.3) days, while that of EBV-positive B cell LAHS or EBV-positive HL LAHS was not evaluable due to limited sample size. Patients whose baseline plasma EBV DNA exceeded 10^5^ copies/mL (median 51 days, 95%CI 30.6-71.4 days) tended to display worse OS compared to patients whose plasma EBV DNA was no more than 10^5^ copies/mL (median 188 days, 95%CI 151.0-225.0 days), but the difference was not significant (*P*=0.159) (**Figure 3A**). ENKL or ANKL patients (median 56 days, 95%CI 42.8-69.2 days) tended to have a poorer OS than patients with other subtypes of lymphoma (not reached), but the difference was not significant (*P*=0.060) (**Figure 3B**). Patients whose baseline ALT exceeded 80 U/L (median 42 days, 95%CI 17.6-66.4 days) had a significantly worse OS than patients with baseline ALT no more than 80 U/L (median 305 days, 95%CI 95.1-514.9 days, *P*=0.041) (**Figure 3C**). EBV-positive LAHS patients who received combined treatment (median 78 days, 95%CI 0.1-230.5 days) had a significantly improved OS compared to patients who only received anti-HLH treatment (median 31 days, 95%CI 18.1-43.9 days) (*P*=0.003) (**Figure 3D**). Specifically, for patients who received combined treatment, those who first received anti-HLH treatment and turned to anti-lymphoma treatment subsequently (median 174 days, 95%CI 0.1-371.9 days) displayed a significantly better OS than patients who only received anti-HLH treatment (median 31 days, 95%CI 18.1-43.9 days, *P*=0.003) (**Figure 3E**). Regarding treatment response, EBV-positive LAHS patients who achieved PR or above (median 305 days, 95%CI 54.2-555.8 days) had a significantly improved OS compared to those who remained NR (median 41 days, 95%CI 31.1-50.9 days, *P*=0.006) (**Figure 3F**). There was no significant influence of other baseline characteristics on OS. For patients who did not receive consolidation treatment, median OS was 56 days (95%CI 28.9-83.1 days). For patients who underwent consolidation treatment, median OS was not reached. Patients who underwent consolidation treatment had a significantly better OS than those without consolidation treatment (HR 0.203, 95%CI 0.048-0.865, *P*=0.031, **Figure S1**). Of the three patients who received PD-1 monoclonal antibody consolidation treatment, one ENKL patient died at 55 days after HLH diagnosis. A DLBCL patient and a HL patient were still alive after a follow-up of 673 days and 1484 days, respectively.

In univariate analysis, lower baseline ALT level, combined treatment of first anti-HLH regimen and subsequent anti-lymphoma regimen, achieving CR or PR were significantly related to improved OS (*P*=0.001, *P*=0.004, and *P*=0.009, respectively). ENKL or ANKL tended to associate with worse OS (*P*=0.069). There was no significant association between plasma EBV-DNA level after treatment and OS. There was no significant relationship between other baseline characteristics and OS. When combining baseline ALT level, combined treatment of first anti-HLH regimen and subsequent anti-lymphoma regimen, treatment response, and ENKL or ANKL subtype in multivariate analysis, elevated baseline ALT level was the independent risk factor of OS (*P*=0.025). Combined treatment of first anti-HLH regimen and subsequent anti-lymphoma regimen and achieving CR or PR were independent protective factors of OS (*P*=0.001, and *P*=0.025, respectively) (**Table 4**).

**Table 4:** Univariate analysis and multivariate analysis of risk factors in EBV-positive LAHS patients.

Factors	Univariate analysis			Multivariate analysis		
	HR	95% CI	*P*	HR	95% CI	*P*
HGB	0.989	0.971-1.006	0.204			
NEUT	0.897	0.763-1.055	0.190			
PLT	0.996	0.987-1.004	0.331			
ALT	1.005	1.002-1.009	0.001	1.007	1.001-1.012	0.025
TG	1.003	0.779-1.290	0.984			
FIB	0.952	0.733-1.234	0.708			
IL6	1.003	1.000-1.006	0.073			
EBV DNA>10^5^ copies/mL	1.769	0.791-3.958	0.165			
ENKL or ANKL	2.458	0.933-2.476	0.069	1.263	0.369-4.326	0.710
anti-HLH to anti-lymphoma v.s. anti-HLH treatment	0.290	0.124-0.676	0.004	0.210	0.081-0.549	0.001
CR or PR v.s. NR	0.358	0.166-0.769	0.009	0.295	0.102-0.857	0.025

ALT, alanine aminotransferase; ANKL, aggressive natural killer cell leukemia; CI, confidence interval; CR, complete response; ENKL, extranodal NK/T-cell lymphoma, nasal type; FIB, fibrinogen; HGB, hemoglobin; HLH, hemophagocytic lymphohistiocytosis; HR, hazard ratio; IL6, interleukin 6; LAHS, lymphoma-associated hemophagocytic syndrome. NEUT, neutrophil; NR, no response; PLT, platelet; PR, partial response; TG, triglyceride.

Regarding EBV-positive T/NK cell LAHS patients, univariate analysis revealed lower ALT level, lower IL6 level, higher PT level, combined treatment of first anti-HLH regimen and subsequent anti-lymphoma regimen, achieving CR or PR were significantly related to improved OS (*P*=0.001, *P*=0.016, *P*=0.044, *P*=0.017, and *P*=0.013, respectively). When combining these factors in multivariate analysis, lower ALT level, combined treatment of first anti-HLH regimen and subsequent anti-lymphoma regimen, and achieving CR or PR were independent protective factors of OS (*P*=0.038, *P*=0.040, and *P*=0.041, respectively).

We also compared prognosis of EBV-positive LAHS patients and EBV-negative LAHS patients in our department. OS of EBV-positive LAHS patients was similar to that of EBV-negative LAHS patients (61 v.s. 42 days, *P*=0.997, **Figure S2**). In univariate analysis of EBV-negative LAHS patients, baseline ferritin more than 2000 ng/mL (*HR*=4.222, 95%CI 1.423-12.528, *P*=0.009), and achieving CR or PR were significantly related to improved OS (*HR*=0.168, 95%CI 0.053-0.536, *P*=0.003). When combining both factors in multivariate analysis, only achieving CR or PR remained significantly related to improved OS (HR=0.228, 95%CI 0.057-0.917, *P*=0.037), serving as protective factor of prognosis.

## Discussion

EBV-positive LPD ranges from reactive proliferation of lymphocytes to lymphoma, and most cases have been reported in East Asia. Due to the tropism of EBV to B cells, EBV-positive LPDs mostly arise from B cell lineage. EBV-positive B-cell lymphoma consist of Burkitt lymphoma, EBV-positive DLBCL in immunocompetent patients, and non-Hodgkin lymphoma associated with human immunodeficiency virus infection in immunodeficient patients. The latent proteins expressed by EBV can immortalize B cell, and the recurrent PD-L1 expression on lymphoma cells indicates an important role of immune evasion in B-cell lymphomagenesis (18, 19). EBV-positive T and NK-cell lymphoma mainly include ENKL, ANKL, systemic EBV-positive T-cell lymphoma of childhood, and primary EBV-positive nodal T and NK-cell lymphoma. The recurrent deletion of LMP1 gene of EBV impairs immune recognition, leading to T and NK-cell lymphomagenesis (20, 21). HLH is a rare hyperinflammatory syndrome triggered by various factors, frequently stimulated by lymphoma or EBV infection. Though some case reports of EBV-positive LAHS have been published, deeper investigation of the disease with a larger sample size is required due to the overlapping clinical manifestations and the poor prognosis. Thus, this study is one of the first and largest cohorts of EBV-positive LAHS patients, aiming at revealing clinical characteristics, management, and prognosis of the disease.

In our study of EBV-positive LAHS patients, T/NK cell lymphoma accounted for 86.3%, while B cell lymphoma only accounted for 9.8%. The proportion of T/NK cell lineage in EBV-positive LAHS was noticeably higher than that in LAHS cohorts of western countries (22), and slightly higher than that in other LAHS cohorts of East Asia (23). This phenomenon could be explained by the following hypothesis. First, ENKL and EBV infection were more prevalent in East Asia than otherwhere. Second, HLH was more commonly triggered by T/NK cell lymphoma than B cell lymphoma. Additionally, it was found in our study that EBV-positive T/NK cell LAHS patients were significantly younger than EBV-positive B cell LAHS patients, similar to the finding of Chang et al. (24). Besides, EBV-positive T/NK cell LAHS patients were found to display significantly lower fibrinogen levels than EBV-positive B cell LAHS patients. We hypothesized that the hyperactivation of T cells in T/NK cell lymphoma might upregulate tissue factors on macrophage surface by generating interferon-gamma, leading to abnormal coagulation activity and fibrinogen consumption (25). Moreover, EBV-positive T/NK cell LAHS patients had significantly lower CRP levels than EBV-positive B cell LAHS patients, which might be attributable to different activation mechanism of monocyte/macrophage lineage in T/NK cell LAHS and B cell LAHS (26).

EBV-associated HLH and EBV-positive LAHS share overlapping clinical manifestations and lab tests, making differential diagnosis thorny and the lymphoma background underestimated. Previous studies have reported that the majority of LAHS manifested itself as HLH upon disease onset, without definite history or evidence of lymphoma (27). In our study, HLH occurred prior to or at the same time of lymphoma in 84.3% of EBV-positive LAHS patients. Thus, for secondary HLH patients, a thorough screening of the potential lymphoma background is needed, even if EBV infection has been confirmed. PET/CT can serve as a sensitive tool to screen lymphoma. It was reported that FDG uptake in LAHS patients was significantly higher than that in other subtypes of HLH patients, and that the level of FDG uptake was related to the extent of cytokine release storm, rendering FDG uptake as a potential predictor of LAHS prognosis (28). Consequently, PET/CT is recommended as a useful tool to search for underlying triggers of secondary HLH.

Due to lack of prospective studies in LAHS treatment, whether initial induction treatment should first focus on HLH or lymphoma has been controversial. Some experts claimed that controlling inflammation should be the priority, because LAHS patients mostly died of MODS caused by cytokine storm. The anti-lymphoma treatment brought about immunosuppression, which might aggravate infection, activate lymphocytes, and amplify cytokine storm (29, 30). Others believed that anti-lymphoma treatment should be initiated immediately with proper anti-inflammatory treatment. Otherwise, HLH might aggravate or relapse rapidly (31). In our study, all EBV-positive LAHS patients received anti-viral treatment immediately. For induction treatment, patients who first underwent anti-HLH treatment for a median time of three weeks and turned to anti-lymphoma treatment subsequently had a significantly better OS and tended to show a higher ORR than patients who only received anti-HLH treatment. This finding indicates that anti-lymphoma therapy should be initiated immediately after cytokine storm is rapidly controlled. Furthermore, diagnosis of lymphoma relies on tissue biopsy. For HLH patients, time should not be wasted on waiting for verification of pathological diagnosis. HLH-1994 regimen and other anti-cytokine storm treatment should be initiated immediately. Individualized lymphoma treatment could be conducted after pathological diagnosis is confirmed. Some studies also claimed that adding doxorubicin or liposomal doxorubicin to the HLH-1994 regimen could control both cytokine storm and lymphoma in the meantime. R-DED (ruxolitinib-doxorubicin-etoposide-dexamethasone) regimen and DEP (liposomal doxorubicin-etoposide-methylprednisolone) regimen yielded significantly higher ORR than the traditional HLH-1994 regimen (23, 32). Thus, it is recommended that anti-HLH treatment should be initiated first and anti-lymphoma treatment should be conducted immediately after cytokine storm is controlled. Prospective studies with larger sample size are still required to explore the optimal induction regimen and strategy. On the other hand, immune evasion has been reported to participate in pathogenesis of EBV-positive HL and EBV-positive DLBCL (33). For consolidation therapy, the EBV-positive HL patient and the EBV-positive DLBCL patient received PD-1 monoclonal antibody consolidation treatment and remained alive until now in our study, indicating the potential application of PD-1 immunotherapy to EBV-positive LAHS treatment.

The prognosis of LAHS is poor. Zhou et al. reported that median OS of LAHS patients on HLH-1994 treatment was only 1.5 months (23). Bigenwald et al. also discovered that median OS of NK/T cell LAHS patients was only 40 days (34). In our study, the median OS of EBV-positive LAHS patients was 61 days, similar to the LAHS study of Ishii et al. (30). Studies investigating prognosis predictors of LAHS patients are rare. Higher ECOG score, skin involvement, lower fibrinogen level, jaundice have been reported as risk factors of LAHS prognosis (24, 35, 36). Studies exploring risk factors of EBV-positive LAHS are lacking. Our study revealed that higher ALT level was an independent risk factor of the disease. Patients who received combined treatment and those who achieved CR or PR had a better prognosis.

With the high prevalence of EBV infection and EBV-associated LPD in East Asia, our study is one of the largest cohort studies to explore clinical features, management, and prognosis of the rare but devastating EBV-positive LAHS. Regarding limitations, this is a single-center, retrospective study, which might bring about selection bias and confounders. Second, the follow-up is relatively short, restricting the observation of clinical outcome. Prospective, multi-center studies with larger sample size and longer follow-up is needed to further investigate optimal management and prognosis predictors of the disease in the future.

## Data Availability Statement

The original contributions presented in the study are included in the article/**Supplementary Material**. Further inquiries can be directed to the corresponding author.

## Ethics Statement

The studies involving human participants were reviewed and approved by West China Hospital, Sichuan University. Written informed consent to participate in this study was provided by the participants’ legal guardian/next of kin.

## Author Contributions

Study conception and design: AZ and TN. Acquisition of data: AZ, JY, ML, LL, XG, JW, HL, KS, and YY. Analysis and interpretation of data: AZ, JY, JW, HL, KS, YY, and TN. Statistical analysis: AZ, JY, ML, LL, and XG. Drafting the article: AZ, JY, ML, LL, and XG. Critical revision of the article: JW, HL, KS, YY, and TN. All authors contributed to the article and approved the submitted version.

## Funding

This work was supported by Incubation Program for Clinical Trials (No. 19HXFH030), Achievement Transformation Project (No. CGZH21001), 1.3.5 Project for Disciplines of Excellence, West China Hospital, Sichuan University (No. ZYJC21007), Translational Research Grant of NCRCH (No. 2021WWB03), and China Postdoctoral Science Foundation (No. 2021M692310).

## Conflict of Interest

The authors declare that the research was conducted in the absence of any commercial or financial relationships that could be construed as a potential conflict of interest.

## Publisher’s Note

All claims expressed in this article are solely those of the authors and do not necessarily represent those of their affiliated organizations, or those of the publisher, the editors and the reviewers. Any product that may be evaluated in this article, or claim that may be made by its manufacturer, is not guaranteed or endorsed by the publisher.
