# Supplementary material for: Epstein-Barr Virus-Positive Lymphoma-Associated Hemophagocytic Syndrome: A Retrospective, Single-Center Study of 51 Patients
Source: Front Immunol. 2022 Apr 11;13:882589. doi: 10.3389/fimmu.2022.882589 (PMC9035824; doi:10.3389/fimmu.2022.882589)

Supplementary Material

# Supplementary Tables

**Table S1**. Comparison of baseline characteristics between EBV-positive LAHS patients and EBV-negative LAHS patients.

| Characteristics | EBV+, n (%)  *N*=51 | EBV-, n (%)  *N*=43 | *P* |
| --- | --- | --- | --- |
| Sex |  |  | 0.200 |
| Male | 35 (68.6) | 24 (55.8) |  |
| Female | 16 (31.4) | 19 (44.2) |  |
| Age at HLH diagnosis, median (range) | 37 (13-64) | 42 (13-90) | 0.049 |
| Lymphoma subtype |  |  | 0.143 |
| T/NK cell lymphoma | 44 (86.3) | 30 (69.8) |  |
| Extranodal NK/T-cell lymphoma, nasal type | 22 (43.1) | 18 (41.9) |  |
| Aggressive natural killer cell leukemia | 13 (25.5) | 1 (2.3) |  |
| Peripheral T cell lymphoma, not otherwise specified | 4 (7.8) | 1 (2.3) |  |
| Angioimmunoblastic T cell lymphoma | 3 (5.9) | 1 (2.3) |  |
| Anaplastic large cell lymphoma | 1 (2.0) | 2 (4.7) |  |
| Systemic EBV-positive T-cell lymphoma of childhood | 1 (2.0) | 0 |  |
| SPTL | 0 | 2 (4.7) |  |
| ITCL | 0 | 2 (4.7) |  |
| HSTL | 0 | 1 (2.3) |  |
| Sezary syndrome | 0 | 1 (2.3) |  |
| T-LBL | 0 | 1 (2.3) |  |
| B cell lymphoma | 5 (9.8) | 10 (23.3) |  |
| Diffuse large B cell lymphoma | 4 (7.8) | 9 (20.9) |  |
| Lymphoplasmacytic lymphoma | 1(2.0) | 0 |  |
| FL | 0 | 1 (2.3) |  |
| Hodgkin lymphoma | 2 (3.9) | 3 (7.0) |  |
| Lymphoma stage |  |  | 1 |
| Ⅰ-Ⅱ stage | 3 (5.9) | 3 (7.0) |  |
| Ⅲ-Ⅳ stage | 48 (94.1) | 40 (93.0) |  |
| IPI score |  |  | 0.565 |
| 0-1 | 2 (3.9) | 4 (9.3) |  |
| 2-3 | 28 (54.9) | 22 (51.2) |  |
| 4-5 | 21 (41.2) | 17 (39.5) |  |
| ECOG score |  |  | 0.872 |
| 0-2 | 30 (58.8) | 26 (60.5) |  |
| 3-5 | 21 (41.2) | 17 (39.5) |  |
| Clinical manifestations |  |  |  |
| Fever | 51 (100) | 43 (100) | NA* |
| Splenomegaly | 42 (82.4) | 35 (81.4) | 0.904 |
| Serous effusion | 28 (54.9) | 17 (39.5) | 0.137 |
| Hepatomegaly | 22 (43.1) | 18 (41.9) | 0.901 |
| Edema | 12 (23.5) | 8 (18.6) | 0.561 |
| Jaundice | 10 (19.6) | 14 (32.6) | 0.151 |
| Lab test, median (range) |  |  |  |
| Hemoglobin (g/L) | 96 (44-132) | 85.5(50-151) | 0.008 |
| Neutrophil (×10^9^/L) | 1.53 (0.03-12.58) | 1.44(0.02-21.18) | 0.934 |
| Platelet (×10^9^/L) | 45 (7-264) | 30.5(4-371) | 0.771 |
| Alanine aminotransferase (U/L) | 87 (10-597) | 53.5(4-1269) | 0.811 |
| Aspartate aminotransferase (U/L) | 108 (15-936) | 95(8-1004) | 0.859 |
| Total bilirubin (μmol/L) | 20.55 (5.20-178.90) | 18.8(2-246.8) | 0.412 |
| Albumin (g/L) | 30.55 (19.0-55.0) | 30.8(17.7-57) | 0.520 |
| Lactate dehydrogenase (U/L) | 758.5 (108-3346) | 836(123-4391) | 0.675 |
| Triglyceride (mmol/L) | 2.885 (0.90-7.66) | 2.315(0.78-3.83) | 0.007 |
| Fibrinogen (g/L) | 1.60 (0.50-6.57) | 1.48(0.25-7.29) | 0.915 |
| Ferritin (ng/mL), n=47 | 2799 (314-161681) | 1334(449-32684) | 0.183 |
| sCD25 (pg/mL), n=38 | 8547.5 (1455-36685) | 6819(1310-48370) | 0.327 |
| Hemophagocytosis phenomenon | 24 (47.1) | 24 (55.8) | 0.398 |
| Occurrence order of lymphoma and HLH |  |  | 0.470 |
| HLH prior to lymphoma | 5 (9.8) | 7 (16.3) |  |
| lymphoma prior to HLH | 8 (15.7) | 4 (9.3) |  |
| Simultaneous occurrence | 38 (74.5) | 32 (74.4) |  |

*NA: not available because event was constant.

| Treatment | EBV+ LAHS, n (%)  (N=46) | EBV- LAHS, n (%)  (N=35) |
| --- | --- | --- |
| Combined treatment | 24 (52.2) | 11 (31.4) |
| Anti-HLH regimen prior to anti-lymphoma chemotherapy | 19 (41.3) | 10 (28.6) |
| Anti-lymphoma chemotherapy prior to anti-HLH regimen | 5 (10.9) | 1 (2.9) |
| Anti-HLH treatment | 18 (39.1) | 13 (37.1) |
| Anti-lymphoma treatment | 3 (6.5) | 5 (14.3) |
| Glucocorticoid treatment | 1 (2.2) | 6 (17.1) |

**Table S2**. Comparison of induction treatment between EBV-positive LAHS patients and EBV-negative LAHS patients.

**Table S3**. Comparison of overall response rate between EBV-positive LAHS patients and EBV-negative LAHS patients.

| Treatment | EBV+ LAHS | | | | EBV- LAHS | | | | *P* |
| --- | --- | --- | --- | --- | --- | --- | --- | --- | --- |
|  | CR | PR | NR | ORR | CR | PR | NR | ORR |  |
| Combined treatment | 2 | 12 | 10 | 58.3% | 2 | 6 | 3 | 72.7% | 0.478 |
| Anti-HLH to anti-lymphoma | 2 | 10 | 7 | 63.2% | 2 | 6 | 2 | 80% | 0.431 |
| Anti-lymphoma to anti-HLH | 0 | 2 | 3 | 40% | 0 | 0 | 1 | 0 | 1 |
| Anti-HLH treatment | 0 | 6 | 12 | 33.3% | 1 | 5 | 7 | 46.2% | 0.710 |
| Anti-lymphoma chemotherapy | 2 | 0 | 1 | 66.7% | 0 | 3 | 2 | 60% | 1 |
| Glucocorticoid treatment | 0 | 0 | 1 | 0 | 0 | 1 | 5 | 16.7% | 1 |
| Total | 4 | 18 | 24 | 47.8% | 3 | 15 | 17 | 51.4% | 0.748 |

# Supplementary Figures

**Figure legends**

**Figure S1**. Comparison of OS between patients who underwent consolidation therapy and those who did not receive consolidation therapy.


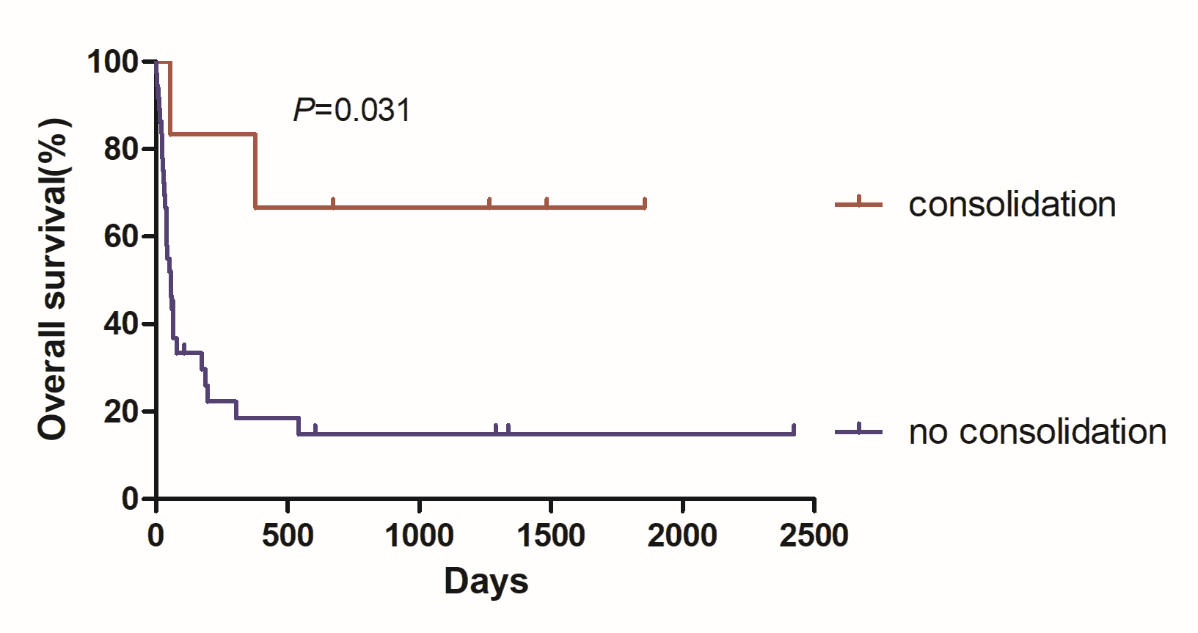


**Figure S2**. Comparison of OS between EBV-positive LAHS patients and EBV-negative LAHS patients.


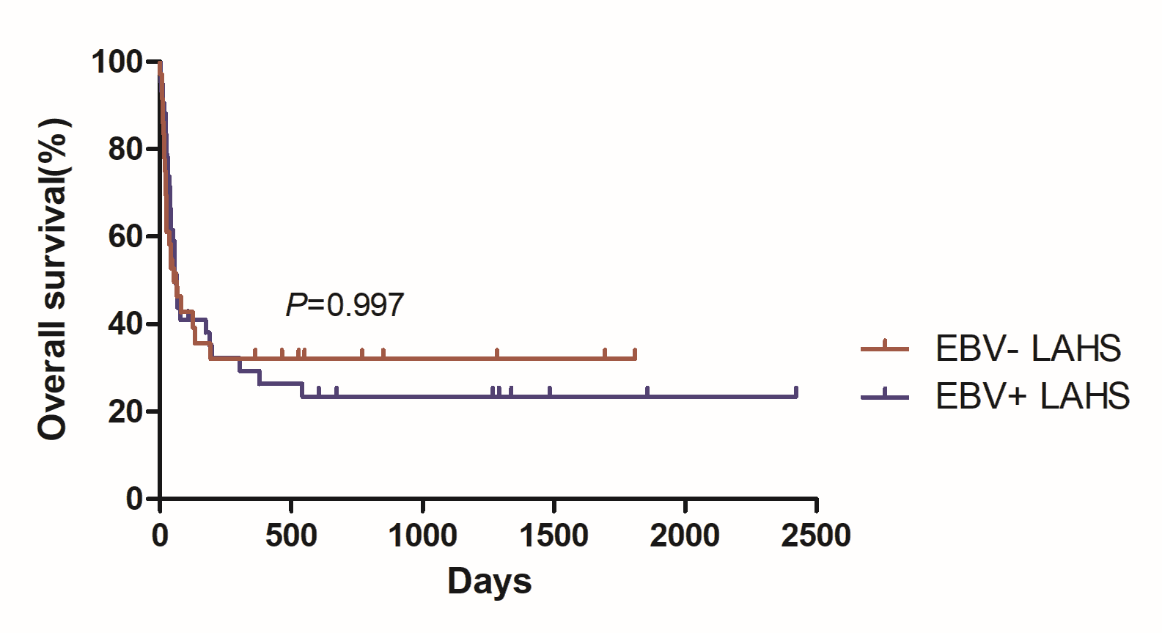

Supplement: Supplementary file 1 [file DataSheet_1.docx]
